# Comparative mitogenomes reveal diverse and novel gene rearrangements in the genus *Meteorus* (Hymenoptera: Braconidae)

**DOI:** 10.3389/fgene.2023.1132606

**Published:** 2023-02-13

**Authors:** Xiaohan Shu, Ruizhong Yuan, Zhilin Xia, Gui Gao, Lei Yang, Zhirong Sun, Qing Mu, Pu Tang, Xuexin Chen

**Affiliations:** ^1^ Hainan Institute, Zhejiang University, Sanya, China; ^2^ Guangdong Laboratory for Lingnan Modern Agriculture, Guangzhou, China; ^3^ State Key Lab of Rice Biology, Ministry of Agriculture Key Lab of Molecular Biology of Crop Pathogens and Insects, and Zhejiang Provincial Key Laboratory of Biology of Crop Pathogens and Insects, Zhejiang University, Hangzhou, China; ^4^ Institute of Insect Sciences, College of Agriculture and Biotechnology, Zhejiang University, Hangzhou, China; ^5^ Guizhou Province Tobacco Companies Qian xinan Municipal Tobacco Company, Xingyi, China; ^6^ Tobacco Leaf Purchase Center, Hunan China Tobacco Industry Co., Ltd., Changsha, China

**Keywords:** Braconidae, *Meteorus*, mitochondrial genome, tRNA gene, gene rearrangement

## Abstract

*Meteorus* Haliday, 1835 is a cosmopolitan genus within Braconidae (Hymenoptera). They are koinobiont endoparasitoids of Coleoptera or Lepidoptera larvae. Only one mitogenome of this genus was available. Here, we sequenced and annotated three mitogenomes of *Meteorus* species, and found that the tRNA gene rearrangements in these mitogenomes were rich and diverse. Compared with the ancestral organization, only seven tRNAs (*trnW*, *trnY*, *trnL2*, *trnH*, *trnT*, *trnP* and *trnV*) were conserved and *trnG* had its own unique location in the four mitogenomes. This dramatic tRNA rearrangement was not observed in mitogenomes of other insect groups before. In addition, the tRNA cluster (*trnA*-*trnR*-*trnN*-*trnS1*-*trnE*-*trnF*) between *nad3* and *nad5* was rearranged into two patterns, i.e., *trnE*-*trnA*-*trnR*-*trnN*-*trnS1* and *trnA*-*trnR*-*trnS1*-*trnE*-*trnF*-*trnN*. The phylogenetic results showed that the *Meteorus* species formed a clade within the subfamily Euphorinae, and were close to *Zele* (Hymenoptera, Braconidae, Euphorinae). In the *Meteorus*, two clades were reconstructed: *M*. sp. USNM and *Meteorus pulchricornis* forming one clade while the remaining two species forming another clade. This phylogenetic relationship also matched the tRNA rearrangement patterns. The diverse and phylogenetic signal of tRNA rearrangements within one genus provided insights into tRNA rearrangements of the mitochondrial genome at genus/species levels in insects.

## 1 Introduction

Braconidae is one of the most species-rich families of Hymenoptera, including 42 subfamilies represented by over 1,100 genera and more than 21,220 known species (Chen and van Achterberg, 2019). *Meteorus* Haliday, 1835 is a cosmopolitan genus within Euphorinae and more than 300 species have been described (Fujie et al., 2021). They are koinobiont endoparasitoids of Coleoptera or Lepidoptera larvae, and some of their hosts are considered pest insects, including some major pests such as *Agrotis ipsilon*, *Helicoverpa armigera*, *Lymantria dispar* and *Spodoptera frugiperda* (Yu et al., 2016). Several final-instar larvae of *Meteorus* can produce cocoons suspended by a common cable, and the cocoon architecture is one of the key characters for identifying these species (Fujie et al., 2021).

The mitochondrial genomes show extremely high rates of gene rearrangements in Hymenoptera compared with other orders in the Hexapoda (Wei et al., 2010; Li et al., 2016). Gene arrangements in the mitochondrial genome can be divided into two types: major rearrangements involving protein-coding genes (PCGs) and rRNAs and minor rearrangements involving tRNAs only based on gene type (Chen et al., 2016). The PCGs rearrangements have been found in Aculeata, Ceraphronoidea, Chalcidoidea, Cynipoidea, Gasteruptiidae, Ichneumonoidea and Trigonaloidea, while the tRNAs rearrangements occur in each family in Hymenoptera (Tang et al., 2019). In addition, rearrangements of the rRNAs have been found in Cynipoidea, Chrysidoidea and Megalyroidea (Tang et al., 2019; Zheng et al., 2021; Shu et al., 2022). In Braconidae, PCGs order is relatively conservative, while tRNA rearrangement patterns within subfamilies have a better taxon representation (Li et al., 2016; Jasso-Martinez et al., 2022a). Such tRNA rearrangement patterns are typically restricted to specific lineages, which can help with phylogenetic reconstruction in Braconidae at the subfamily level (Li et al., 2016).

Here, we re-sequenced the mitogenome of *Meteorus pulchricornis* and newly obtained two other *Meteorus* mitogenomes by next-generation sequencing. We further analyzed the main features of the three mitogenomes. Then we compared gene rearrangements of four known mitogenomes within the genus *Meteorus*. Finally, we confirmed the phylogenetic position of *Meteorus* within Braconidae based on mitogenome data.

## 2 Methods

### 2.1 Sample collection and DNA extraction

*M. pulchricornis*, *M*. sp. 1 and *M.* sp. 2 were all collected in the Chinese provinces of Zhejiang (Ningbo city), Guizhou (Guiyang city), and Hebei (Shijiazhuang city), respectively. The specimens were morphologically identified by Prof. Cornelis van Achterberg (Zhejiang University, China). All specimens were initially preserved in 100% ethanol and then stored at 4 °C before DNA extraction. Whole genomic DNA was extracted from every sample using the DNeasy tissue kit (Qiagen, Hilden, Germany).

### 2.2 High throughput sequencing and assembly

The libraries were prepared for each DNA sample using the VAHTS^®^ Universal DNA Library Prep Kit. All constructed libraries were then sequenced as 150 bp paired-end on a full run (2 × 150 PE) using MGISEQ2000 platform. FastQC v0.11.9 (Andrews, 2015) was used to check the data quality, and fastp v0.23.1 (Chen et al., 2018) was used to trim adaptors and remove low quality reads with default parameters. More than 5 GB of clean data for each sample was used in *de novo* assembly. The mitogenomes were assembled using MitoZ v2.3 (Meng et al., 2019), IDBA v1.1.3 (Peng et al., 2012) and SPAdes v3.13.0 (Bankevich et al., 2012) with default parameters, respectively. Subsequently, to verify the accuracy of *de novo* assembly, one fragment in the *cox1*-*cox2* junction was amplified with primers (C1-J-2195: 5’-TGA​TTT​TTT​GGG​CAT​CCT​GAA​GT-3’, C2-N-3665: 5’-CCA​CAA​ATT​TCA​GAA​CAT​TGA​CC-3’) for three species by polymerase chain reaction (PCR) and was then Sanger-sequenced. Finally, all assemblies were then integrated with GENEIOUS v2020.0.5 (Biomatters Ltd. San Diego, CA, United States).

### 2.3 Mitochondrial genome annotation and analysis

Three assembled mitogenomes were annotated using MITOS web server (http://mitos.bioinf.uni-leipzig.de/index.py; accessed on 15 November 2022) (Bernt et al., 2013). The start and stop positions of 13 PCGs were manually adjusted and corrected by aligning published data of the Braconidae species in GenBank. tRNAScan-SE (Chan et al., 2021) was used to verify the results of putative tRNA genes. Nucleotide composition, codon usage, and relative synonymous codon usage (RSCU) values were estimated using MEGA 11 (Tamura et al., 2021). The bias of nucleotide composition was measured as AT-skew = (A − T)/(A + T) and GC-skew = (G − C)/(G + C) (Perna and Kocher, 1995). The gene rearrangements in *Meteorus* mitogenomes were analyzed by comparison with the ancestral insect mitogenomes (Boore, 1999).

### 2.4 Phylogenetic analysis

A total of 21 Braconidae mitogenomes were used for phylogenetic analyses, including three newly obtained mitogenomes. Two Ichneumonidae species, *Euceros kiushuensis* and *Diadegma semiclausum*, were used as outgroups (Supplementary Table S1). The PCGs were aligned using MAFFT v7.464 (Katoh and Standley, 2013) with the default algorithm, and the best partition schemes and substitution models (Supplementary Table S2) for the datasets were analyzed by PartitionFinder v1.1.1 (Lanfear et al., 2012). Bayesian inference (BI) and maximum likelihood (ML) were selected to reconstruct the phylogenetic trees. For BI analysis, MrBayes v3.2.7a (Ronquist and Huelsenbeck, 2003) was used to run four independent Markov chains for 100 million generations, with tree sampling occurring every 1,000 generations and a burn-in of 25% of the trees. The stationarity of the run was assessed by Tracer v1.7. (ESS values >200) (Rambaut et al., 2018). Maximum likelihood (ML) analysis was performed with RAxML-HPC2 v8.2.12 (Stamatakis, 2014) under the GTRGAMMA model. A total of 200 runs for different individual partitions were conducted with 1,000 bootstrap replicates.

## 3 Results and discussion

### 3.1 Mitochondrial genome organization

**FIGURE 1:**
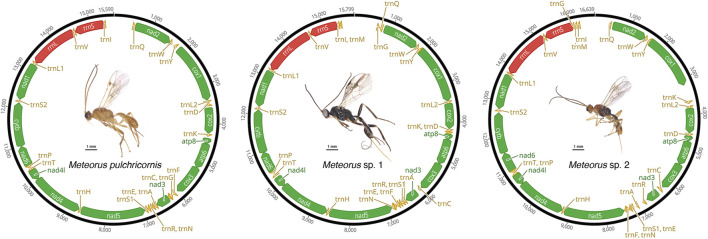
Mitochondrial maps of three *Meteorus* species.

The mitogenomes of three *Meteorus* species were successfully obtained, and the newly sequenced mitogenomes were submitted to GenBank (accession numbers: OP832526 - OP832528) (Supplementary Table S1). All sequences were nearly complete mitogenomes, measuring 15,590 bp for *M. pulchricornis*, 15,799 bp for *M*. sp. 1, and 16,639 bp for *M.* sp. 2. In all assembled mitogenomes, all 13 protein-coding genes (PCGs), 22 tRNA genes, and two rRNA genes were found (Figure 1). Non-etheless, the A-T control region was incomplete in all genomes due to the high A+ T content in the Hymenoptera mitogenomes (Zheng et al., 2021; Jasso-Martinez et al., 2022a).

The mitogenome of *M. pulchricornis* contained 19 non-coding regions ranging in size from 1 to 67 bp, with a total length of 407 bp. The nucleotides from 8 overlap regions were up to 22 bp in total. The maximum overlap length was 7 bp, located at two junctions (*atp8*-*atp6*, *nad4*-*nad4l*), while the minimum overlap length was 1 bp and occurred at four junctions (*atp6*-*cox3*, *trnA*-*trnR*-*trnN*, *trnT*-*trnP*). *M*. sp. 1 had 18 non-coding regions with lengths ranging from 1 to 83 bp, for a total of 303 bp. The nucleotides from 5 overlap regions amounted to 18 bp in total. Two junctions (*atp8*-*atp6*, *nad4*-*nad4l*) had overlaps of 7 bp in length. *M*. sp. 2 possessed 23 non-coding regions ranging from 1 to 465 bp in length and a total length of 1,138 bp, which had the longest intergenic nucleotides among the three *Meteorus* mitogenomes. It had 3 overlap regions with a total of 12 bp in length. Three junctions (*nad4*-*nad4l*, *atp8*-*atp6*, and *trnE*-*trnF*) had overlaps of 7 bp, 4 bp and 1 bp in length, respectively. The length of overlap (4 bp) at the junction *atp8*-*atp6* in the mitogenomes of *M*. sp. 2 was unusually short compared to that of other wasps, which was typically 7 bp (Zhu et al., 2018; Tang et al., 2019).

### 3.2 Nucleotide composition

The A + T content for the sequenced region of the mitogenomes was 84.41% (*M. pulchricornis*), 82.70% (*M.* sp. 1) and 84.31% (*M.* sp.2) (Supplementary Table S3), which was similar to other wasp species (Tang et al., 2019). All 13 PCGs were detected in the newly generated mitogenomes, with sizes ranging from 11,063 bp (*M.* sp. 1) to 11,120 bp (*M.* sp. 2). The entire A + T content of all the PCGs was from 80.48% (*M.* sp. 1) to 83.37% (*M. pulchricornis*) (Supplementary Table S3). The size of the PCGs in the three mitochondrial genomes was similar to other wasps (Zheng et al., 2021; Shu et al., 2022; Zheng et al., 2022). The AT-skew in three *Meteorus* mitogenomes was negative (−0.1574 to −0.1479), and the GC-skew was positive (0.0662–0.1088) (Supplementary Table S3). All 22 typical tRNA genes were found in three mitogenomes. The size of all tRNA genes identified ranged from 57 bp to 72 bp (Supplementary Table S4). Two rRNA genes (*rrnS* and *rrnL*) were identified in all mitogenomes. The length of *rrnS* was 764 bp (*M. pulchricornis*), 735 bp (*M*. sp. 1) and 790 bp (*M*. sp. 2), and the size of *rrnL* was 1,253 bp, 1,325 bp and 1,347 bp, respectively (Supplementary Table S3).

### 3.3 Protein-coding genes and codon usage

**FIGURE 2:**
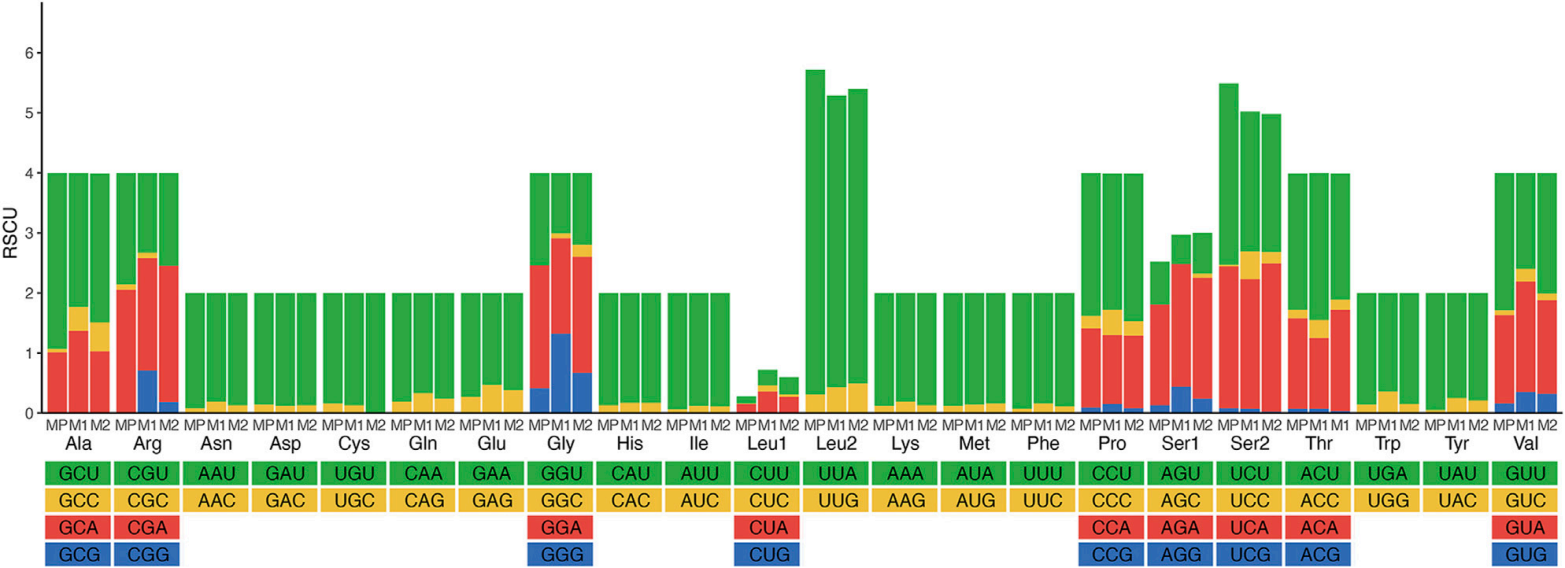
Relative synonymous codon usage (RSCU) of three *Meteorus* species. Codon families are provided on the *X*-axis along with the different combinations of synonymous codons that code for that amino acid. RSCU is defined on the *Y*-axis. MP, *Meteorus pulchricornis*; M1, *Meteorus* sp. 1; M2, *Meteorus* sp. 2.

The majority of the protein-coding genes used ATN (ATG, ATT, or ATA), except ATC, as an initiation codon. Although most PCGs terminated with the conventional TAA and TAG as stop codons, some of them have incomplete stop codons (T or TA) in some PCGs (Supplementary Table S5). The start and stop codons were typical of insect mitogenomes (Li et al., 2021; Ge et al., 2022). Relative synonymous codon usage (RSCU) values of three *Meteorus* species were analyzed (Figure 2; Supplementary Table S6). The total codon number of these three species was 3,689, 3,677 and 3,695, respectively. The codon GCG was not detected in all species, while codons UGC, CGC, AGC and GGC appeared in some species. Meanwhile, the five most frequently used codons UUA, AUU, UUU, AUA and AAU were observed in the mitogenomes due to the high A + T content in the mitogenomes. These results are consistent with other published wasp mitogenomes (Zhu et al., 2018; Zheng et al., 2022).

### 3.4 Gene rearrangement

**FIGURE 3:**
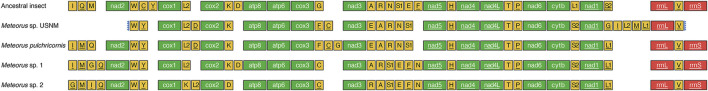
Mitochondrial gene order in *Meteorus* genus and ancestral insect. Genes are transcribed from left to right except those underlined, which have the opposite transcriptional orientation.

The order of PCGs was relatively conservative in the mitogenomes of Braconidae. So far, only *Stenocorse bruchivora* (Doryctinae), two *Chelonus* spp. (Cheloninae), and two *Cotesia* spp. (Microgastrinae) were found PCGs rearrangement in mitogenomes (Wei et al., 2010; Jasso-Martinez et al., 2022a; Yuan et al., 2022). In contrast, tRNA rearrangements occurred in all known Braconidae mitogenomes (Li et al., 2016; Jasso-Martinez et al., 2022a). PCG rearrangements did not occur in any of the four *Meteorus* mitogenomes studied, but tRNA rearrangements varied (Figure 3). Only seven tRNAs (*trnW*, *trnY*, *trnL2*, *trnH*, *trnT*, *trnP* and *trnV*) were conserved and *trnG* had different locations in each of the four mitogenomes. *trnL1* and *trnS2* were inverted and exchanged. Furthermore, *trnC* was translocated from *nad2*-*cox1* junction to *cox3*-*nad3* junction. The tRNA cluster (*trnA*-*trnR*-*trnN*-*trnS1*-*trnE*-*trnF*) between *nad3* to *nad5* exhibited two distinct patterns. In *M.* sp. USNM and *M. pulchricornis*, *trnE* was translocated upstream of *trnA*, and *trnF* was rearranged to *cox3*-*nad3* junction, which reduced one tRNA in the tRNA cluster. In *M.* sp. 1 and *M.* sp. 2, the number of tRNAs between *nad3* to *nad5* remained at six, and *trnN* was translocated downstream of *trnF*. In addition, the positions of *trnA* and *trnR* were interchanged in *M.* sp. 2. Interestingly, *trnI* and *trnM*, which were usually conservative in Braconidae mitogenomes, were translocated from upstream of *nad2* to the *nad1*-*rrnL* junction, and *trnL2* was duplicated into the *nad1*-*rrnL* junction as well in *M.* sp. USNM. In summary, the pattern of tRNAs suggested that *M.* sp. USNM and *M. pulchricornis* were more closely related than the other two species.

The rich and diverse rearrangements presented by the tRNAs in the genus *Meteorus* were the first to be found in the Braconidae. tRNA rearrangements are usually conserved in the same genus in Hymenoptera (Jasso-Martinez et al., 2022a). Although different rearrangement of tRNAs within the same genus in Hymenoptera has been reported in Aphelinidae, Chrysididae and Ichneumonidae (Zhu et al., 2018; Zheng et al., 2021; Zheng et al., 2022), the tRNA rearrangements within *Meteorus* in Braconidae were more dramatic than in the aforementioned families, which exhibited only one or two different tRNA rearrangements in the same genus. To our knowledge, the various tRNA rearrangement patterns within the same genus had not been found in the other insects.

### 3.5 Phylogenetic analysis

**FIGURE 4:**
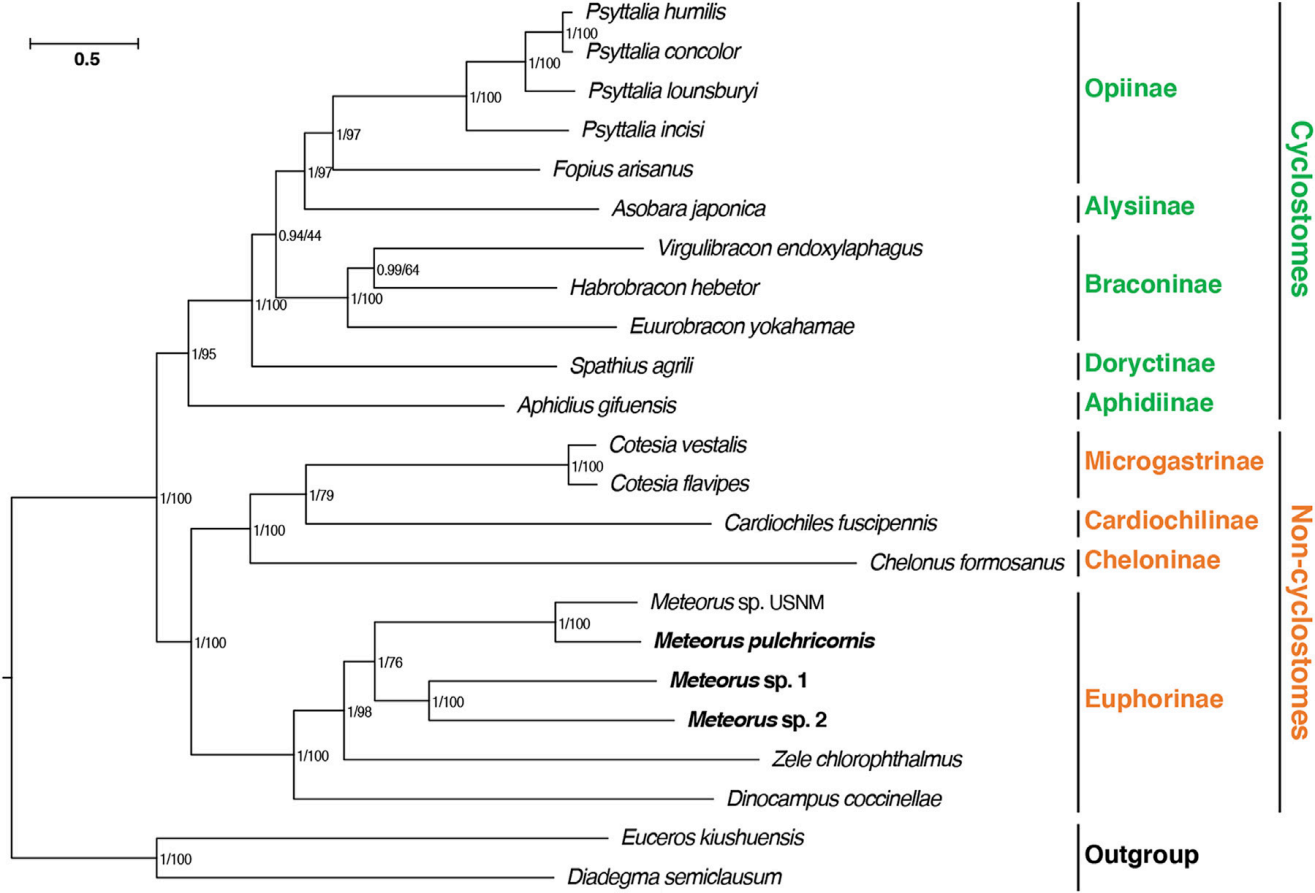
Phylogenetic analyses of Braconidae based on nucleotide datasets of 13 PCGs. The scale bar corresponds to the estimated number of substitutions per site. Numbers separated by a slash on the node are posterior probability (PP) and bootstrap value (BV).

In this study, a phylogenetic analysis based on 13 PCGs was constructed using maximum likelihood and Bayesian methods, in order to validate the phylogenetic position of *Meteorus* within Braconidae (Figure 4). The consensus tree derived from the above two inference methods generated the congruent results. Most nodes had significantly supported bootstrap and posterior probability values. The tree recovered the well-accepted major lineages within the family and supported the division of Braconidae into cyclostomes and non-cyclostomes as generally accepted (Jasso-Martinez et al., 2022a; Jasso-Martinez et al., 2022b). Meanwhile, consistent with previous studies, within the non-cyclostomes, Euphorinae was recovered as a sister clade to the remaining non-cyclostomes (Jasso-Martinez et al., 2022b). The four *Meteorus* species formed a clade within Euphorinae, and were close to *Zele chlorophthalmus* rather than *Dinocampus coccinellae*. In the *Meteorus*, the species were clustered in two clades: one comprised of *M*. sp. USNM and *M. pulchricornis* while *M*. sp. 1 and *M*. sp. 2 grouped together in the second clade. This structure matched the tRNA rearrangement result presented above.

## 4 Conclusion

In this study, three *Meteorus* mitogenomes were newly acquired using next-generation sequencing method, which expanded the data of mitochondrial genomes in the subfamily Euphorinae. Three mitogenomes of *Meteorus* spp. shared similar A + T content, AT- and GC-skew, and codon usage of PCGs, however, *M.* sp. 2 had longer intergenic nucleotides. Each mitogenome among the *Meteorus* spp. displayed dramatic divergent gene rearrangements within the same genus that had not previously been reported. The pattern of tRNAs between *nad3* to *nad5* had two types, *trnE*-*trnA*-*trnR*-*trnN*-*trnS1* and *trnA*-*trnR*-*trnS1*-*trnE*-*trnF*-*trnN*. The BI and ML analyses showed consistent topology, *Meteorus* species formed a clade within Euphorinae, and were close to *Zele*. Two clades were reconstructed for the *Meteorus*, which matched the tRNA rearrangement patterns. In the future, more mitochondrial genomes from the same genus are needed to display further details of tRNA rearrangements. The diverse and phylogenetic signal of tRNA rearrangements within one genus may provide insights into the potential mechanism of tRNA rearrangements in the same genus and the phylogenetic relationships of taxa at the genus level.

## Data Availability

The datasets presented in this study can be found in online repositories. The names of the repository/repositories and accession number(s) can be found below: https://www.ncbi.nlm.nih.gov/, OP832526—OP832528.
